# Synergistic treatment of sodium propionate and Sishen Pill for diarrhea mice with kidney-yang deficiency syndrome

**DOI:** 10.3389/fcimb.2025.1608271

**Published:** 2025-06-02

**Authors:** Meifang Guo, Jiaxin Di, Zhijun Lei

**Affiliations:** School of Pharmacy, Hunan University of Chinese Medicine, Changsha, Hunan, China

**Keywords:** Sishen Pill, sodium propionate, kidney-yang deficiency syndrome, intestinal microorganisms, enzyme activity

## Abstract

**Objective:**

This study aimed to investigate the therapeutic effects of sodium propionate in combination with Sishen Pill in the treatment of diarrhea with kidney-yang deficiency syndrome in mice, with a focus on its influence on intestinal microbiota, enzyme activity, and associated therapeutic outcomes.

**Methods:**

A model of diarrhea with kidney-yang deficiency syndrome was established via adenine combined with *Folium sennae* administration. The model group was randomly assigned to the following treatment groups: natural recovery group, 100% Sishen Pill group, 75% Sishen Pill + 60 mg/kg sodium propionate group, 50% Sishen Pill + 120 mg/kg sodium propionate group, 25% Sishen Pill + 240 mg/kg sodium propionate group, and 480 mg/kg sodium propionate group. A variety of parameters, including general symptoms, body weight, rectal temperature, intestinal microbiota composition, microbial activity, and enzyme activity, were assessed.

**Results:**

Compared with natural recovery group, the 480 mg/kg sodium propionate group presented significant improvements in mental state, anal temperature, fecal water content, the thymus index, and *Bifidobacterium* counts (*p* < 0.01). Compared with those in normal group, the fecal water content and *Escherichia coli* counts in the 100% Sishen Pill group were significantly different (*p* < 0.01), but no significant differences were observed compared with those in natural recovery group (*p* > 0.05). The 75% Sishen Pill + 60 mg/kg sodium propionate group showed improvements in mental state, food and water intake, body weight, rectal temperature, fecal water content, spleen, and thymus index, *Bifidobacterium* counts, total bacterial count, *E. coli* count, microbial activity, and lactase activity, which were close to normal levels, and significant differences were observed when compared to the natural recovery group (*p* < 0.01). The 50% Sishen Pill + 120 mg/kg sodium propionate group and 25% Sishen Pill + 240 mg/kg sodium propionate group also exhibited significant differences in mental status, microbial activity, and Lactobacillus count relative to those of normal group (*p* < 0.01). Furthermore, the 50% Sishen Pill + 120 mg/kg sodium propionate group presented significant changes in fecal water content (*p* < 0.01), whereas 25% Sishen Pill + 240 mg/kg sodium propionate group presented significant differences in the spleen index, total bacterial count, *E. coli* count, protease activity, lactase activity, and xylanase activity compared with those of normal group (*p* < 0.01).

**Conclusion:**

The 75% Sishen Pill + 60 mg/kg sodium propionate can improve the symptoms of kidney-yang deficiency syndrome, promote the growth and development of mice, inhibit excessive bacterial proliferation, support the growth of beneficial bacteria, and enhance intestinal enzyme activity. Its effects are superior to the use of sodium propionate or Sishen Pill alone. These results suggest that this therapeutic ratio may optimize the efficacy of Sishen Pill in the treatment of diarrhea with kidney-yang deficiency syndrome. However, further research is necessary to confirm whether this combination represents the most effective treatment regimen for this condition in mice.

## 1 Introduction

Kidney-yang deficiency syndrome is primarily caused by insufficient kidney-yang, leading to the gradual deterioration of spleen and stomach function, which weakens or disrupts digestive processes (Zhang et al., 2017). Diarrhea with kidney-yang deficiency syndrome is a common condition in traditional Chinese medicine (TCM) and is typically characterized by an increased frequency of loose stools, often accompanied by symptoms such as lower back and knee soreness, as well as cold intolerance and cold extremities (Sun et al., 2023). Contemporary microbiological research has revealed a strong correlation between the onset of diarrhea and an imbalance in the intestinal microbiota. Dysbiosis of the intestinal microbiota can disrupt various host functions, including the loss of barrier integrity, increased inflammation, and impaired immune responses, all of which can predispose individuals to disease (Li et al., 2021c). Patients with irritable bowel syndrome-diarrhea (IBS-D) related to spleen and kidney yang deficiency also present with intestinal microbiota disturbances, such as reduced levels of *Lactobacillus* and *Bifidobacterium*, as well as elevated levels of *Escherichia coli* and *Enterococcus*. The interplay between diarrhea with kidney-yang deficiency syndrome and intestinal microbiota dysbiosis is bidirectional: diarrhea with kidney-yang deficiency syndrome exacerbates the imbalance in the intestinal microbiota, which, in turn, further aggravates the condition of diarrhea with kidney-yang deficiency syndrome.

Propionate, a short-chain fatty acid (SCFA), has been shown to promote the growth of beneficial bacteria and regulate intestinal microecological balance (Howard et al., 2022). The upregulation of inflammatory mediators such as Tumor Necrosis Factor Alpha (TNF-α) and Interleukin 6 (IL-6) increases endothelial cell permeability, leading to the leakage of intestinal fluids and the onset of diarrhea (Chen et al., 2022). Furthermore, the dysregulated expression of numerous inflammatory factors, once released into the bloodstream, circulates to the intestines, resulting in intestinal wall congestion and edema. Subsequently, ischemia and hypoxia in the intestinal tissue compromise the mucosal barrier, exacerbating dysbiosis (Peng et al., 2021). Propionate functions as a nonsteroidal anti-inflammatory agent, exerting its effects primarily through the inhibition of cyclooxygenase enzymes in the arachidonic acid metabolic pathway (Yuenyongviwat et al., 2021). In addition, propionate modulates intestinal motility, decreasing peristalsis, which aids in controlling diarrhea (Song et al., 2022). Studies have also revealed a correlation between the characteristic intestinal microbiota species *Lactobacillus johnsonii* and propionate in mice with kidney-yang deficiency syndrome following intervention with the Sishen Pill (Li et al., 2023). Moreover, propionate can regulate the balance of the intestinal microbiota and facilitate the recovery of intestinal function, which is particularly important in intestinal dysfunction resulting from kidney yang deficiency.

Sishen Pill, a classical formula in traditional Chinese medicine, consists of six key ingredients: *Psoralea corylifolia L.*, *Myristica fragrantis*, *Euodia rutaecarpa*, *Schisandra chinensis*, *Zingiber officinale*, and *Ziziphus jujuba*. It has the functions of warming the kidneys, dispersing cold, and astringing the intestines to stop diarrhea, making it particularly effective for conditions related to kidney yang deficiency (Zhou et al., 2011). In previous studies conducted by our research group, we demonstrated that Sishen Pill intervention in a mouse model of diarrhea with kidney-yang deficiency syndrome significantly improved renal structure, energy metabolism, and the diversity and composition of the intestinal microbiota (Zhu et al., 2023). After Sishen Pill treatment, we observed substantial reductions in the relative abundances of Firmicutes and *Mycoplasma* species, accompanied by notable increases in *Clostridium*, *Turicibacter*, and *Romboutsia* species (Liu et al., 2019). These results suggest that Sishen Pill exerts a modulatory effect on the intestinal microbiota, promoting the growth of beneficial bacteria, reducing pathogenic bacteria, and helping restore the balance of the intestinal microbiota in the host.

The use of drug combinations has become increasingly prevalent in recent research. For example, polysaccharide components from *Ganoderma lucidum* and triterpene compounds from *Chrysanthemum* indicum, both of which exhibit antitumor activity, are capable of inducing mitochondrial damage and apoptosis in human leukemia 60 cells to varying degrees. When these drugs are administered together, their apoptotic effects are significantly potentiated (Kim et al., 2007). Sodium propionate functions primarily to regulate the intestinal microbiota, with a limited impact on kidney tonification and yang enhancement. In contrast, Sishen Pill is recognized for its ability to warm the kidneys, dispel cold, and astringe the intestines to alleviate diarrhea, making it particularly relevant for treating kidney-yang deficiency. Therefore, this experiment aims to combine sodium propionate with Sishen Pill, hoping to maintain the intestinal microecological balance and promote nutrient absorption through the action of sodium propionate, thereby indirectly guiding the absorption and utilization of the kidney-tonifying and yang-enhancing components in Sishen Pill, and achieving a synergistic and amplifying therapeutic effect.

Based on the aforementioned findings, this study established a mouse model of diarrhea with kidney-yang deficiency syndrome, which aligns with the etiology and pathogenesis described in TCM. This model provides a strong foundation for the development of formulations that combine sodium propionate and Sishen Pill. The results offer valuable insights for investigating the relationship between intestinal homeostasis and overall health, as well as for elucidating the therapeutic mechanisms underlying the synergistic effects of TCM herbal combinations.

## 2 Materials and methods

### 2.1 Materials

#### 2.1.1 Animals

To eliminate the influence of sex on the intestinal microbiota, this study used only male mice, whose intestinal microbiota has been widely validated in multiple studies. This makes them an ideal choice for studying intestinal microbiota changes related to sex hormones and immune responses, especially due to their good compatibility with the kidney-yang deficiency syndrome model (Wu et al., 2022). A cohort of 70 Kunming SPF-grade male mice, each weighing between 18 and 22 grams and aged 4 weeks, was procured from Huna Slake Jinda Experimental Animal Mal Co., Ltd. (China, Animal Quality Certificate No. ZS-202404230013). Moreover, this study was approved by the Animal Ethics Committee of Hunan University of Chinese Medicine and was conducted in compliance with ethical standards (Ethics No. SLBH-202404130001).

#### 2.1.2 Drugs

Adenine (Changsha Yale Bio-Tech Co., Ltd., Batch No. EZ7890B179) and *Folium sennae* (Haozhou Luqiao Pharmaceutical Co., Ltd., Batch No. 2111090022) were utilized in this study. The formulation of Sishen Pill included 12 g of *Psoralea corylifolia L.* (Hunan Hengyue Traditional Chinese Medicine Decoction Pieces Co., Ltd., Batch No. 231001), 6 g of *Myristica fragrantis* (Hunan Hengyue Traditional Chinese Medicine Decoction Pieces Co., Ltd., Batch No. 23083107), 3 g of *Euodia rutaecarpa* (Hunan Rongkang Traditional Chinese Medicine Decoction Pieces Co., Ltd., Batch No. 230801), 6 g of *Schisandra chinensis* (Haozhou Yonggang Decoction Pieces Co., Ltd., Batch No. A231031), 6 g of *Ziziphus jujuba* (Hunan Xinshenzhilin Traditional Chinese Medicine Decoction Pieces Co., Ltd., Batch No. 2310001), and 6 g of *Zingiber officinale* (Xie et al., 2024).

The adenine suspension was prepared as follows: adenine was dissolved to achieve a concentration of 5 mg/mL and was freshly prepared as needed (Fang et al., 2025). Similarly, in preparation for Senna decoction, *Folium sennae* were immersed in 300 mL of water for 30 minutes. The mixture was subsequently boiled and simultaneously boiled for an additional 30 minutes. The solution was then filtered through gauze, and the residue was re-extracted with 200 mL of water via the same procedure. The 2 filtrates were combined and concentrated to yield a Senna decoction with a final concentration of 1 g/mL, which was stored at 4°C for future use (Li et al., 2023). Similarly, in the preparation of Sishen Pill decoction, the solution was concentrated to 0.29 g/mL via a method analogous to that employed for Senna decoction (Li et al., 2023). This decoction was then diluted to 25%, 50%, 75%, and 100% of the original Sishen Pill decoction concentration. The prepared solutions were stored at 4°C for future use.

#### 2.1.3 Reagent

Fluorescein Diacetate (Shanghai Yuan Ye Biotechnology Co., Ltd.); Sodium Chloride (National Group Chemical Reagents Co., Ltd.); Potassium Chloride (National Group Chemical Reagents Co., Ltd.); Disodium Hydrogen Phosphate (National Group Chemical Reagents Co., Ltd.); Potassium Dihydrogen Phosphate (National Group Chemical Reagents Co., Ltd.); Acetone (Hunan Huihong Reagents Co., Ltd.); Citric Acid (Mudanjian City Fengda Chemical Import & Export Co., Ltd.); Sodium Citrate (Langfang Pengcai Fine Chemical Co., Ltd.); Soluble Starch (Tianjin Damo Chemical Reagents Factory); Dodecahydrate Disodium Hydrogen Phosphate (National Group Chemical Reagents Co., Ltd.); Dihydrate Potassium Dihydrogen Phosphate (National Group Chemical Reagents Co., Ltd.); Sodium Hydroxide (Xilong Chemical Co., Ltd.); Trichloroacetic Acid (National Group Chemical Reagents Co., Ltd.); Sodium Carbonate (National Group Chemical Reagents Co., Ltd.); Xylan (Shanghai Yuan Ye Biotechnology Co., Ltd.); 2-Nitrophenyl-β-D-Galactopyranoside (Shanghai Yuan Ye Biotechnology Co., Ltd.); Nitrophenol (Shanghai Xiechen Import & Export Co., Ltd.); Folin Phenol (Shanghai Xinyu Biotechnology Co., Ltd.); Sodium Tartrate (National Group Chemical Reagents Co., Ltd.); 3,5-Dinitrosalicylic Acid (Shanghai Runcheng Biotechnology Co., Ltd.); Trihydrate Disodium Hydrogen Phosphate (National Group Chemical Reagents Co., Ltd.); Heptahydrate Magnesium Sulfate (National Group Chemical Reagents Co., Ltd.); Ethylenediaminetetraacetic Acid (National Group Chemical Reagents Co., Ltd.); Fluorescein Diacetate (Shanghai Yuan Ye Biotechnology Co., Ltd.); Beef Extract (Beijing Solebao Technology Co., Ltd.); Peptone (Beijing Aoboxing Biotechnology Co., Ltd.); Agar Powder (Beijing Lanjieke Technology Co., Ltd.); Lactose (Tianjin Kemiou Chemical Reagents Co., Ltd.); Potassium Hydrogen Phosphate (National Group Chemical Reagents Co., Ltd.); Eosin Methylene Blue (Shanghai Shengsi Biochemical Technology Co., Ltd.); Yeast Extract (Beijing Solebao Technology Co., Ltd.); Glucose (Tianjin Baishi Chemical Co., Ltd.); Cysteine (Shanghai Maclin Biochemical Technology Co., Ltd.); Tween (Changsha Deyu Plastics Co., Ltd.).

Sodium propionate (Shanghai Aladdin Biochemical Technology Co., Ltd., Batch No. A2431246) was dissolved in sterile water to prepare solutions with concentrations of 480 mg/(kg•d), 240 mg/(kg•d), 120 mg/(kg•d), and 60 mg/(kg•d). These solutions were subsequently stored at 4°C in a refrigerator until use (Song et al., 2022).

#### 2.1.4 Culture media

**Total Bacteria (Beef Extract Peptone Agar Medium):** Add 5 g of beef extract, 10 g of peptone, 5 g of NaCl, and 15 g of agar powder into a triangular flask, then add 1 L of distilled water. Stir with a glass rod to dissolve thoroughly and adjust the pH of the solution to 7.2-7.4.; ***Escherichia coli* (Eosin Methylene Blue Agar Medium):** Add 10 g of peptone, 10 g of lactose, 2 g of K2HPO4, 15 g of agar, 0.4 g of eosin, and 0.065 g of methylene blue into a triangular flask, then add 1 L of distilled water. Stir with a glass rod to dissolve thoroughly and adjust the pH of the solution to 7.0-7.2; ***Bifidobacterium* (BBL Agar Medium):** Add 15 g of peptone, 2 g of yeast extract, 20 g of glucose, 0.5 g of soluble starch, 5 g of NaCl, 10 mL of 5% cysteine, 520 mL of distilled water, 400 mL of tomato extract, 1 mL of Tween 80, 80 mL of liver extract, and 20 g of agar into a triangular flask. Stir with a glass rod to dissolve thoroughly and adjust the pH of the solution to 6.9-7.1; ***Lactobacillus* (MRS Agar Medium):** Add 7.5 g of yeast extract, 7.5 g of peptone, 10 g of glucose, 2 g of K2HPO4, 100 mL of tomato juice, 0.5 g of Tween 80, 15 g of agar into a triangular flask, then add 900 mL of distilled water. Stir with a glass rod to dissolve thoroughly and adjust the pH of the solution to 7.0 (Peleman et al., 2017).

#### 2.1.5 Experimental facilities for feed and shielding environments

This study was con ducted with support from the Animal Experimentation Center of Hunan University of Chinese Medicine (Shen et al., 2025).

### 2.2 Methods

#### 2.2.1 Modeling methods

The animal model of diarrhea with kidney-yang deficiency syndrome was established following the protocol outlined in the literature (Guo et al., 2024; Xie et al., 2024). The animal model of diarrhea with kidney-yang deficiency syndrome was established using adenine combined with *Folium sennae*. The model group was administered a daily gavage of adenine suspension at a dose of 50 mg/(kg•d), with a volume of 0.35 mL per mouse, for 14 consecutive days. Starting on day 8, the mice were additionally administered a senna leaf decoction at a dose of 10 g/(kg·d), 0.35 mL per mouse, once daily for 7 consecutive days. The normal group was given an equivalent volume of sterile water following the same administration schedule. Successful modeling was confirmed when the mice presented characteristic symptoms, including loose stools or incomplete fecal pellets, cold extremities, an arched posture, a reduced appetite, weight loss, and lethargy.

#### 2.2.2 Grouping and method of administration

After the successful establishment of the model, the modeling factors were discontinued. The model groups were then randomly assigned to the following treatment groups using a random number table: natural recovery group and treatment groups (100% Sishen Pill, 75% Sishen Pill + 60 mg/kg sodium propionate, 50% Sishen Pill + 120 mg/kg sodium propionate, 25% Sishen Pill + 240 mg/kg sodium propionate, and 480 mg/kg sodium propionate) (Song et al., 2022). Each group consisted of 10 animals. 100% Sishen Pill group: Gavage with a water decoction of Sishen Pill at an equivalent dose of 5 g/(kg·d). 75% Sishen Pill + 60 mg/kg sodium propionate group: Gavage with 75% Sishen Pill water decoction + 60 mg/(kg·d) sodium propionate. 50% Sishen Pill + 120 mg/kg sodium propionate group: Gavage with 50% Sishen Pill water decoction + 120 mg/(kg·d) sodium propionate. 25% Sishen Pill + 240 mg/kg sodium propionate group: Gavage with 25% Sishen Pill water decoction + 240 mg/(kg·d) sodium propionate. 480 mg/kg sodium propionate group: Gavage with 480 mg/(kg·d) sodium propionate. Each treatment group received 0.35 mL per animal, twice daily, for 7 consecutive days. The normal group and natural recovery group were gavaged with the same number of times and volume of sterile water.

#### 2.2.3 General observation of experimental animal health

After the successful establishment of the model and the completion of the treatment, the mental status, fecal consistency, and behavioral changes of the experimental animals were observed. Modeling phase: Body weight, rectal temperature, and diarrhea index of the mice were measured and recorded on days 1 and 14. Water consumption and food intake were measured and recorded on days 1, 7, and 13 of the modeling phases (Tian et al., 2021). Treatment phase: Body weight, rectal temperature, and the diarrhea index were subsequently measured and recorded on days 2, 4, and 7, whereas water consumption and food intake were recorded on days 1, 4, and 7.

#### 2.2.4 Detection of immune organs in mice

Upon completion of the treatment, the mice were weighed, and the weights of the spleen and thymus were measured. The organ indices were subsequently calculated via the following formulas: Spleen Index (%) = (Spleen Weight (g)/Mouse Body Weight (g)) × 100%; Thymus Index (%) = (Thymus Weight (g)/Mouse Body Weight (g)) × 100%.

#### 2.2.5 Intestinal microbial activity in mice

Samples of murine intestinal contents were collected and diluted with sterile water at a ratio of 3 g to 50 mL. The resulting mixture was then shaken on an orbital shaker for 90 minutes to ensure adequate dispersion of the microorganisms. After centrifugation, the supernatant was carefully collected. The absorbance values at 490 nm were measured in triplicate for each sample; the A490 value indicates the microbial activity per unit mass of the sample (Zhou et al., 2023a).

#### 2.2.6 Determination of the number of intestinal microorganisms

Aseptic techniques were utilized to transfer a specified volume of intestinal content into a sterile bottle containing glass beads. The resulting mixture was subsequently agitated in a shaking incubator at 120 r/min for 30 minutes to ensure comprehensive dispersion of the microorganisms. After this step, appropriate dilution factors were selected, and a mixed culture counting method was employed. Aerobic bacteria and coliform bacteria were cultivated in a 37°C incubator for 24 hours before colony enumeration, whereas *Lactobacillus* and *Bifidobacterium* were incubated anaerobically at 37°C for 48 hours before enumeration. Each dilution was conducted in triplicate to derive an average value, and using manual counting, the colony count on each plate is recorded for plates with a colony count between 30 and 300, and the number of colony-forming units (CFU/g) per gram of sample is calculated based on the dilution factor and inoculation volume (Wu et al., 2021).

#### 2.2.7 Analysis of intestinal enzyme activity in mice

The intestinal contents from each group were placed into sterile centrifuge tubes containing an appropriate amount of sterile water and 7–8 glass beads. The samples were vortexed for 2 minutes using a vortex shaker to facilitate the complete release of enzymatic substances. The mixture was then centrifuged at 3000 rpm and 4°C for 10 minutes, and the supernatant was collected as crude enzyme solution. For enzyme activity measurement, to ensure experimental accuracy and comparability, one blank control tube and three sample tubes were set up for each experiment. Lactase and protease activities were measured using the o-Nitrophenyl-β-D-galactopyranoside (ONPG) method and the Folin-phenol method, respectively. Xylanase and amylase activities were determined using the DNS colorimetric method (Wu et al., 2021).

#### 2.2.8 Statistical analysis

The data were analyzed via SPSS version 25.0. One-way analysis of variance (ANOVA) was performed under the assumptions of normality and homogeneity of variance. In instances where these assumptions were violated, nonparametric tests for multiple samples were utilized. The significance level of *p* < 0.05 was considered statistically significant, whereas *p* < 0.01 was considered highly significant.

## 3 Results and analysis

### 3.1 Modeling diarrhea in kidney-yang deficiency syndrome and the effects of the combination of sodium propionate and Sishen Pill on symptoms and signs in mice

**Figure 1:**
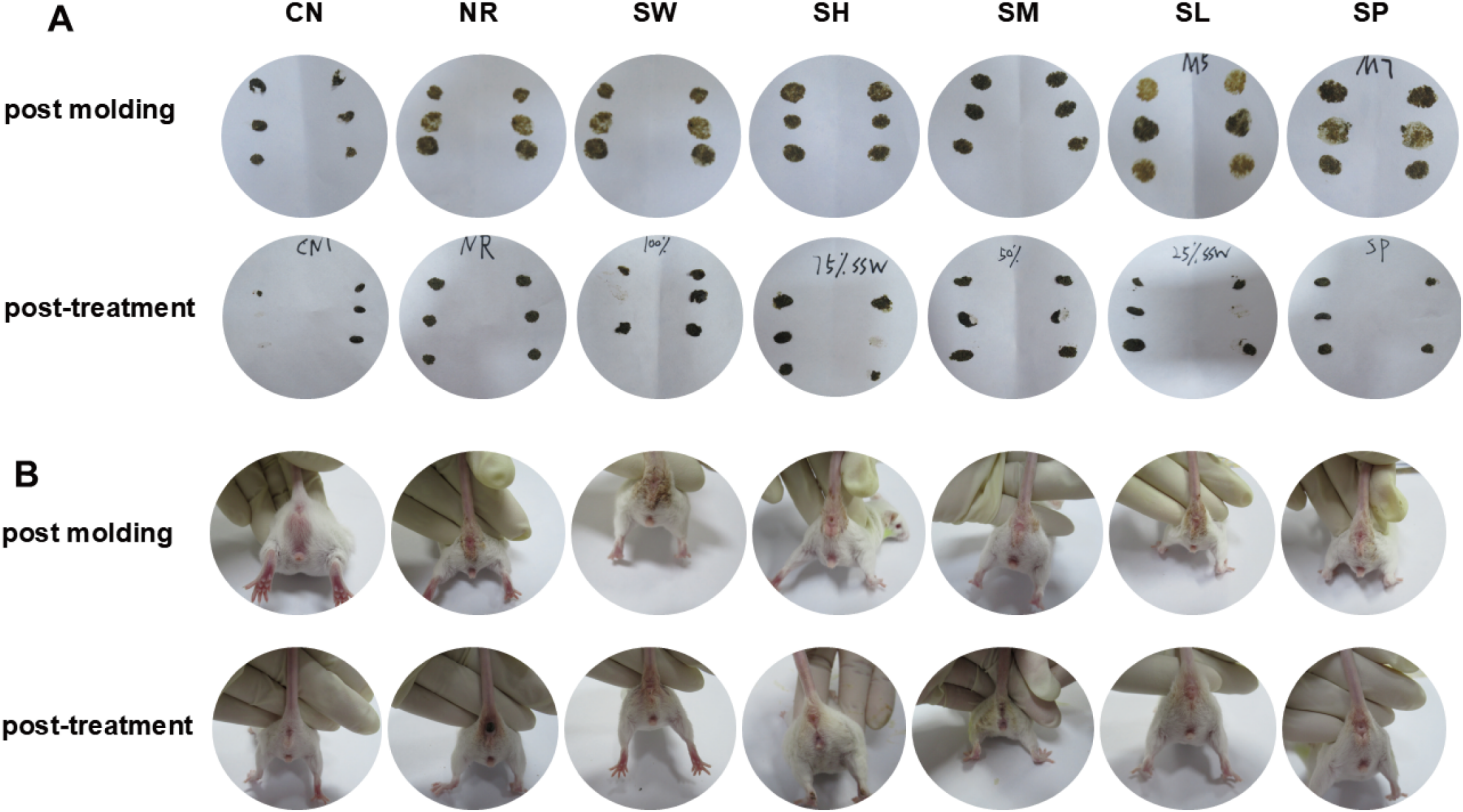
Modeling diarrhea in kidney-yang deficiency syndrome and the effects of combining sodium propionate with Sishen Pill on **(A)** fecal characteristics and **(B)** perianal cleanliness in mice. The following experimental groups were established: CN: normal group; NR: natural recovery group; SW: 100% Sishen Pill group; 75% Sishen Pill + 60 mg/kg sodium propionate: 75% Sishen Pill + 60 mg/kg sodium propionate group; SM: 50% Sishen Pill + 120 mg/kg sodium propionate group; SL: 25% Sishen Pill + 240 mg/kg sodium propionate group; SP: 480 mg/kg sodium propionate group. The groups are the same as the image below.

During the treatment period, we systematically observed the behavioral performance of the mice, and the results are shown in **Figures 1A,B**. Significant alterations were noted in the viscosity of feces, mental state, and fur luster before and after the intervention. On day 1 of treatment, the freshly excreted feces remained loose and soft, accompanied by wet bedding, perianal soiling, lethargy, drowsiness, reduced responsiveness, hunching, clustering, and a noticeable lack of luster in the fur. In contrast, on day 7 of treatment, the 100% Sishen Pill group, the 75% Sishen Pill + 60 mg/kg sodium propionate group, and the 480 mg/kg sodium propionate group exhibited pronounced therapeutic effects, with a marked alleviation of symptoms of diarrhea with kidney-yang deficiency syndrome. Additionally, both the 50% Sishen Pill + 120 mg/kg sodium propionate group and the 25% Sishen Pill + 240 mg/kg sodium propionate group demonstrated varying degrees of improvement throughout the treatment duration.

### 3.2 Modeling diarrhea in kidney-yang deficiency syndrome and the effects of the combination of sodium propionate and Sishen Pill on body weight in mice

**Figure 2:**
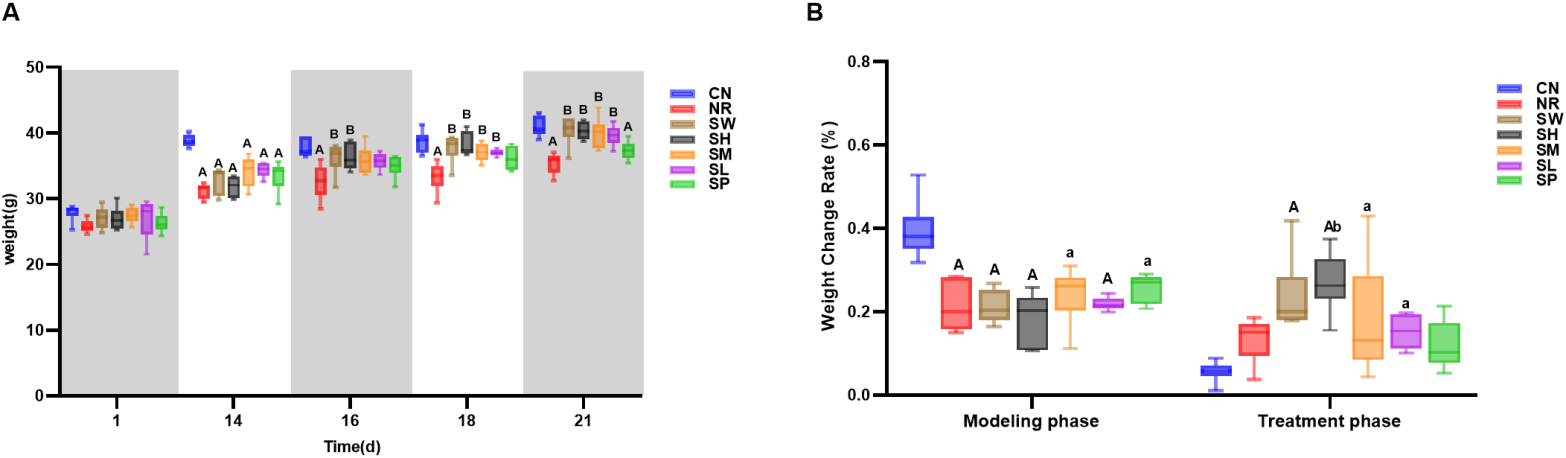
Modeling diarrhea in kidney-yang deficiency syndrome and the effects of combining sodium propionate with Sishen Pill on the body weight **(A)** and body weight change rate **(B)** of mice (x̄ ± s, N=6). Compared with CN, a: *p* < 0.05, A: *p* < 0.01; compared with NR, b: *p* < 0.05, B: *p* < 0.01; compared with SW, c: *p* < 0.05, C: *p* < 0.01; compared with SL, d: *p* < 0.05, D: *p* < 0.01; compared with SM, e: *p* < 0.05, E: *p* < 0.01; compared with SH, f: *p* < 0.05, F: *p* < 0.01, same below.

According to the results in **Figure 2A**, the weight changes of mice in all groups were similar during the adaptive feeding period. After 14 days of modeling, the body weight of mice in all 7 experimental groups was higher than their initial weight. The body weight of mice in model group was significantly lower than that of normal group (*p* < 0.05). As the number of days of intervention with sodium propionate and Sishen Pill increased, the body weight of natural recovery group remained significantly lower than that of normal group (*p* < 0.01), and the rate of weight change in natural recovery group was not significantly different from normal group (*p* > 0.05) (**Figures 2A,B**). After the treatment ended, the body weight of mice in the 100% Sishen Pill group, 75% Sishen Pill + 60 mg/kg sodium propionate group, 50% Sishen Pill + 120 mg/kg sodium propionate group, and 25% Sishen Pill + 240 mg/kg sodium propionate group was close to that of the normal group (*p* > 0.05), and all showed significant differences when compared to the natural recovery group (*p* < 0.01). Among them, the weight change rate in the 75% Sishen Pill + 60 mg/kg sodium propionate group was significantly higher than that in normal and natural recovery groups (*p* < 0.05). In contrast, the body weight of mice in the 480 mg/kg sodium propionate group was significantly lower than that of normal group (*p* < 0.01), and the rate of weight change was not significantly different from normal group (**Figures 2A,B**). These results indicate that the aqueous extract of Sishen Pill can promote weight gain in diarrhea mice with kidney-yang deficiency syndrome to a certain extent, gradually approaching the normal level, with the best effect observed in the 75% Sishen Pill + 60 mg/kg sodium propionate combination. At the same time, sodium propionate did not show a significant effect on increasing the body weight of diarrhea mice with kidney-yang deficiency syndrome.

### 3.3 Modeling diarrhea in kidney-yang deficiency syndrome and the effects of combining sodium propionate with Sishen Pill on rectal temperature and diarrhea index in mice

**Figure 3:**
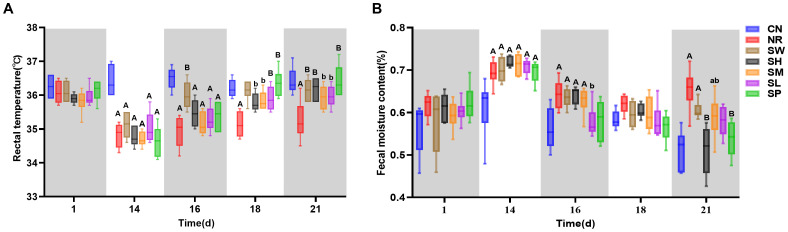
Modeling Diarrhea in Kidney-Yang Deficiency Syndrome and the Effects of Combining Sodium Propionate with Sishen Pill on the Rectal Temperature **(A)** and Diarrhea Index **(B)** of mice (x̄ ± s, N=6).

The observations presented in **Figure 3A** clearly show that, on day 1 of modeling, there was a minimal difference in rectal temperature between the normal group and the model groups. However, by day 14 of the modeling period, the rectal temperatures of the mice in the model groups were significantly lower than those of normal group (*p* < 0.01). This finding indicates that the model for diarrhea with kidney-yang deficiency syndrome results in a decrease in rectal temperature. On day 2 of treatment (day 16 of the experiment), the increase in rectal temperature in 100% Sishen Pill group was more pronounced than that in natural recovery group (*p* < 0.01). On day 7 of treatment (day 21 of the experiment), the rectal temperatures of the mice across all the treatment groups approached the levels observed in normal group; the temperature of natural recovery group remained significantly lower than that of normal group. These results suggest that intervention with Sishen Pill effectively elevates rectal temperature in mice suffering from diarrhea with kidney-yang deficiency syndrome, progressively restoring it to normal levels. Moreover, higher concentrations of Sishen Pill are correlated with more rapid recovery, whereas the influence of sodium propionate appears to be minimal.

As shown in **Figure 3B**, upon the completion of the experimental model, model group presented significantly greater fecal water contents than normal group (*p* < 0.01). This observation confirms the successful induction of diarrhea in these mice. On day 2 of treatment (day 16 of the experiment), the diarrhea indices for the natural recovery and treatment groups gradually decreased, with the indices for 25% Sishen Pill + 240 mg/kg sodium propionate and 480 mg/kg sodium propionate groups approaching those of normal group (*p* > 0.05). By the 7th day of treatment (day 21 of the experiment), the diarrhea indices of the 75% Sishen Pill + 60 mg/kg sodium propionate and 480 mg/kg sodium propionate groups were significantly lower than those of natural recovery group (*p* < 0.01) and were comparable to the levels observed in normal group. In contrast, the diarrhea indices of the natural recovery, 100% Sishen Pill, and 50% Sishen Pill + 120 mg/kg sodium propionate groups remained significantly greater than those of normal group (*p* < 0.01 or *p* < 0.05). These results indicate that both 75% Sishen Pill + 60 mg/kg sodium propionate in conjunction with sodium propionate, as well as sodium propionate administered alone, are effective at reducing the diarrhea index in mice with kidney yang deficiency syndrome, thereby restoring it to normal physiological levels.

### 3.4 Modeling diarrhea in kidney-yang deficiency syndrome and the effects of the combination of sodium propionate and Sishen Pill on food intake and water intake in mice

**Figure 4:**
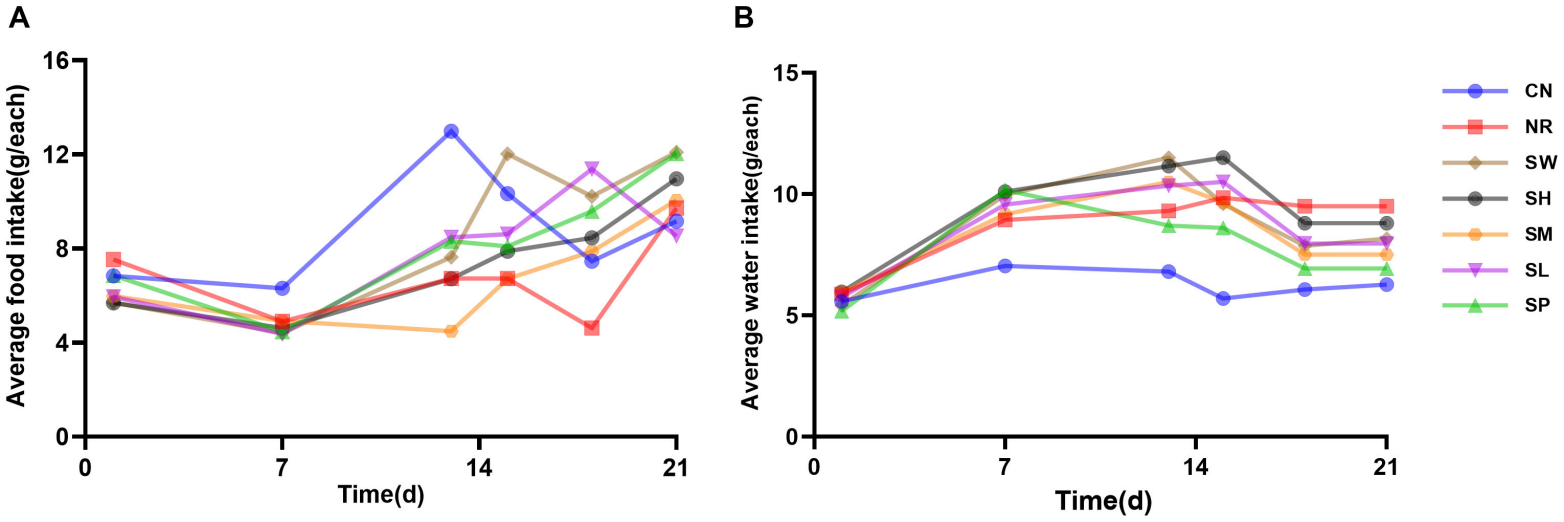
Modeling diarrhea in kidney-yang deficiency syndrome and the effects of combining sodium propionate with Sishen Pill on food intake **(A)** and water intake **(B)** in mice (x̄ ± s, N=10).

As illustrated in **Figure 4**, the daily average water intake of normal group of mice showed little fluctuation; however, the daily average food intake showed significant variability. Throughout the modeling phase, the daily average food intake across model groups remained lower than that of normal group. The average water intake of the mice in the other six groups showed a progressively stable increase compared to the normal group. These findings indicate that during the 14-day modeling process, the administration of adenine combined with *Folium sennae* led to a decrease in the average food intake while simultaneously increasing the average water intake of the mice.

During the treatment phase, fluctuations in food intake among the 7 groups remained pronounced as the duration of treatment increased. The daily average food intake of natural recovery group of mice continued to be lower than that of normal group, recovering only by day 7. In contrast, the treatment groups exhibited relatively rapid normalization of food intake. Notably, the daily average water intake of the treatment group was greater than that of normal group; however, it generally decreased compared to that of natural recovery group. These findings suggest that the treatment group reduced the water intake of diarrhea with kidney-yang deficiency syndrome to near-normal levels, although its efficacy in improving food consumption was limited.

### 3.5 Effects of sodium propionate and Sishen Pill on immune organs in diarrhea mice with kidney-yang deficiency syndrome

**Figure 5:**
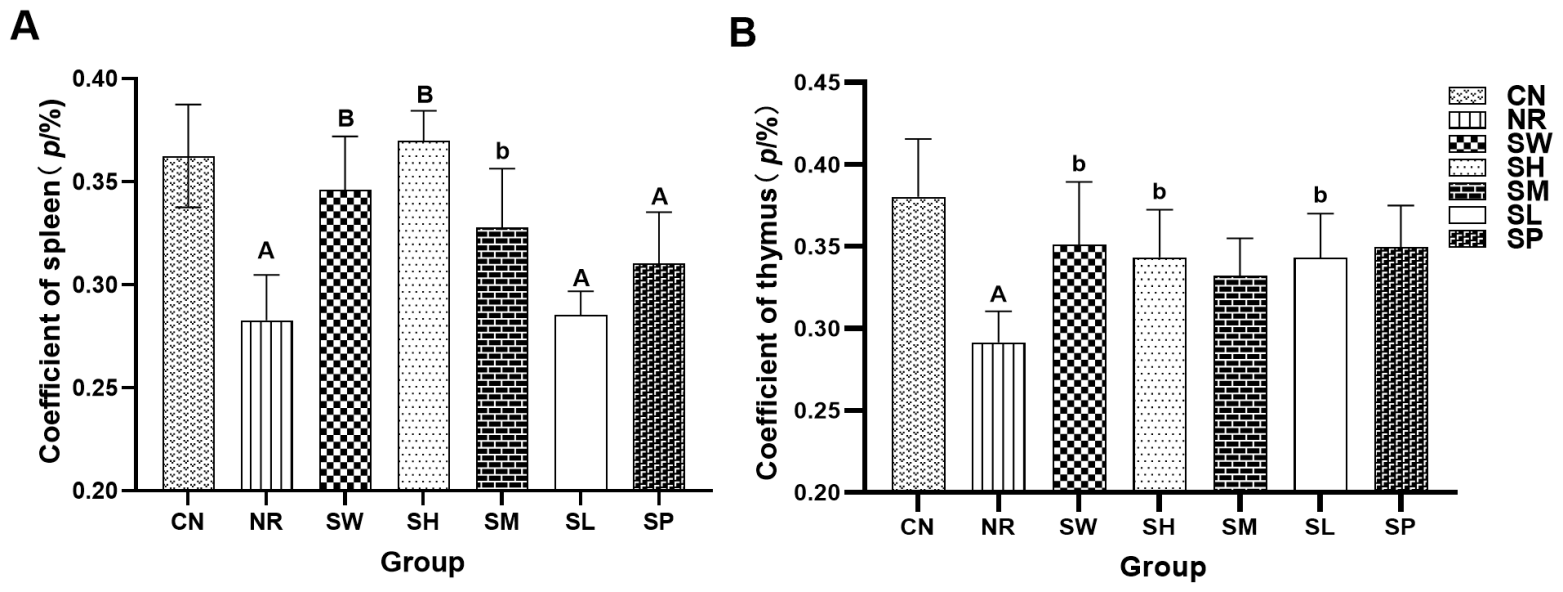
Effects of sodium propionate and Sishen Pill on immune organs in diarrhea mice with kidney-yang deficiency syndrome (x̄ ± s, N=6). **(A)** Coefficient of the spleen. **(B)** Coefficient of the thymus index. Compared with CN, a: p < 0.05, A: p < 0.01; compared with NR, b: p < 0.05, B: p < 0.01.

As depicted in **Figure 5**, the spleen indices of natural recovery group, 25% Sishen Pill + 240 mg/kg sodium propionate, and 480 mg/kg sodium propionate groups were significantly lower (*p* < 0.01) than those of normal group. The thymus index of normal group was markedly greater than that of natural recovery group (*p* < 0.01). Notably, the spleen indices of the 100% Sishen Pill, 75% Sishen Pill + 60 mg/kg sodium propionate, and 50% Sishen Pill + 120 mg/kg sodium propionate groups were significantly greater than those of natural recovery group (*p* < 0.01 or *p* < 0.05). Furthermore, the thymus indices of the 100% Sishen Pill, 75% Sishen Pill + 60 mg/kg sodium propionate, and 480 mg/kg sodium propionate groups were significantly greater than those of natural recovery group (*p* < 0.05). These findings indicate that the administration of adenine combined with *Folium sennae* via gavage has a notable inhibitory effect on the development of the spleen and thymus in mice. Additionally, Sishen Pill can enhance the physiological functions of mice after inducing diarrhea with kidney-yang deficiency syndrome. Notably, sodium propionate seems to increase the thymus index; however, its relationship with the spleen index does not appear to be statistically significant.

### 3.6 Effects of sodium propionate and Sishen Pill on intestinal microorganisms in diarrhea mice with kidney-yang deficiency syndrome

**Figure 6:**
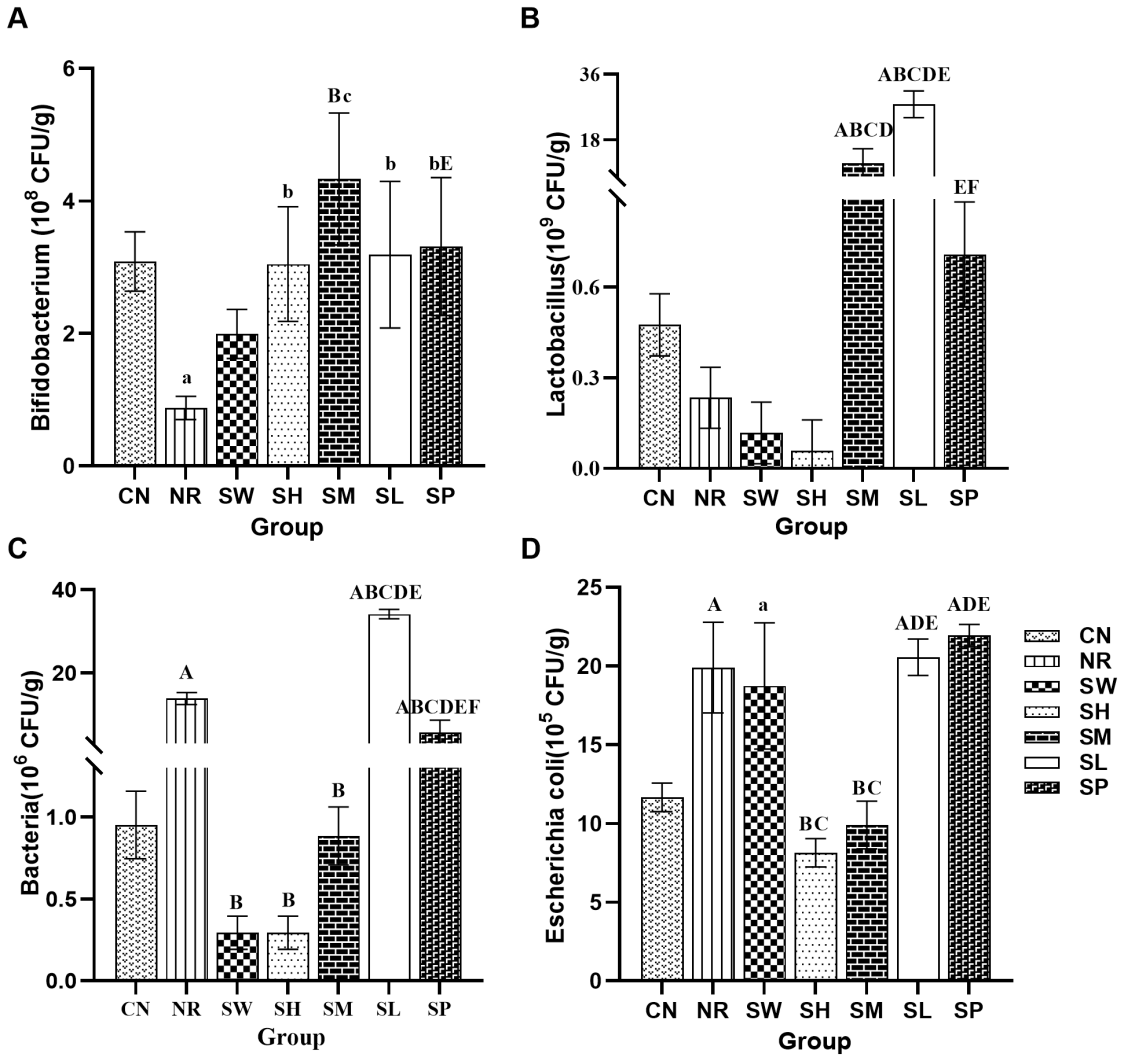
Effects of sodium propionate and Sishen Pill on intestinal microorganisms in diarrhea mice with kidney-yang deficiency syndrome (x̄ ± s, N=6). **(A)** *Bifidobacterium*; **(B)** *Lactobacillus*; **(C)** Total bacterial count; **(D)** *Escherichia coli*.

As shown in **Figure 6**, the levels of *Bifidobacterium* in natural recovery group were significantly lower than those in normal group (p < 0.01). No statistically significant differences (*p* > 0.05) were observed between the remaining groups and normal group, indicating that the modeling method effectively inhibited the growth of *Bifidobacterium*, whereas the treatment groups promoted its proliferation.

Regarding *Lactobacillus* abundance, no significant differences (*p* > 0.05) were found between normal group, natural recovery group, 100% Sishen Pill group, 75% Sishen Pill + 60 mg/kg sodium propionate group, and 480 mg/kg sodium propionate group. However, both 50% Sishen Pill + 120 mg/kg sodium propionate group and 25% Sishen Pill + 240 mg/kg sodium propionate group exhibited a significant increase in *Lactobacillus* abundance compared to both normal and natural recovery groups (*p* < 0.01). This suggests that while the modeling method, as well as the administration of 75% Sishen Pill + 60 mg/kg sodium propionate and sodium propionate alone, had minimal effects on *Lactobacillus*, the combination of lower and medium doses of Sishen Pill with sodium propionate promoted the growth of *Lactobacillus*.

In terms of the total bacterial count, a significant increase in the total intestinal microbiota count was observed in natural recovery group, 25% Sishen Pill + 240 mg/kg sodium propionate group, and 480 mg/kg sodium propionate group compared with normal group (*p* < 0.01). In contrast, no statistically significant differences (*p* > 0.05) were detected among the other groups. This finding indicates that the modeling method may lead to an increase in total bacterial counts, with the 100% Sishen Pill, 75% Sishen Pill + 60 mg/kg sodium propionate, and 50% Sishen Pill + 120 mg/kg sodium propionate groups effectively inhibiting bacterial growth. In contrast, the 25% Sishen Pill + 240 mg/kg sodium propionate and 480 mg/kg sodium propionate groups exhibited the opposite effect.

In terms of *Escherichia coli*, a significant increase in *E. coli* counts was noted in natural recovery group, 100% Sishen Pill group, 25% Sishen Pill + 240 mg/kg sodium propionate group, and 480 mg/kg sodium propionate group relative to normal group (*p* < 0.01). No statistically significant differences (*p* > 0.05) were observed in the remaining groups. These findings suggest that the modeling method can increase the number of *E. coli*, with 75% Sishen Pill + 60 mg/kg sodium propionate and 50% Sishen Pill + 120 mg/kg sodium propionate groups effectively suppressing bacterial growth. In contrast, the 100% Sishen Pill, 25% Sishen Pill + 240 mg/kg sodium propionate, and 480 mg/kg sodium propionate groups presented opposite effects.

### 3.7 Effects of sodium propionate and Sishen Pill on the activity of intestinal microorganisms in diarrhea mice with kidney-yang deficiency syndrome

**Figure 7:**
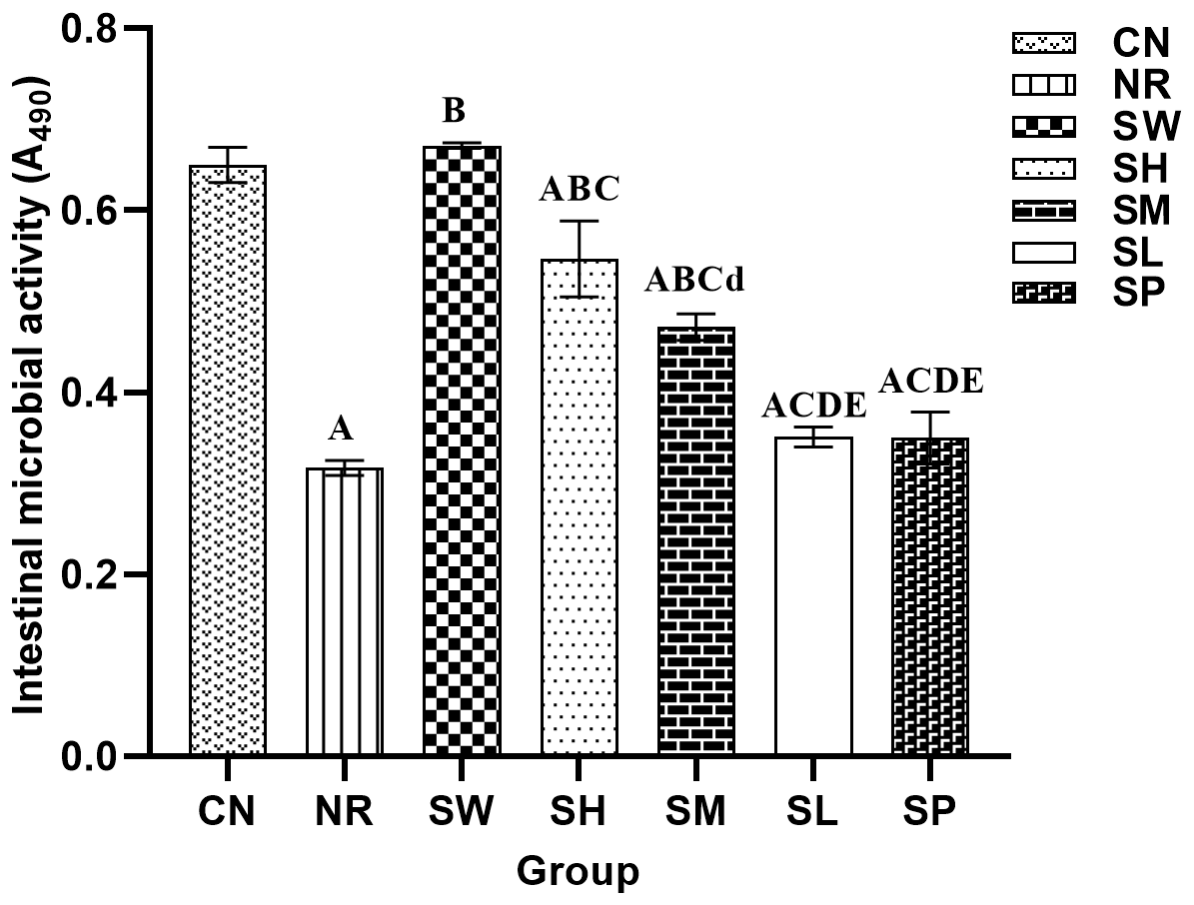
Effects of sodium propionate and Sishen Pill on the activity of intestinal microorganisms in diarrhea mice with kidney-yang deficiency syndrome (x̄ ± s, N=6).

As shown in **Figure 7**, the microbial activity in natural recovery group was significantly lower than in normal group (*p* < 0.01). The administration of adenine combined with senna resulted in a marked decrease in intestinal microbial activity in mice, suggesting that the modeling process may have disrupted the metabolic functions of the microbiota and inhibited the growth of associated microorganisms. Furthermore, in comparison with those in natural recovery group, significant increases in microbial activity were observed in the 100% Sishen Pill, 75% Sishen Pill + 60 mg/kg sodium propionate, and 50% Sishen Pill + 120 mg/kg sodium propionate groups (*p* < 0.01). As the concentration of Sishen Pill increased, the intestinal microbial activity in the mice progressively approached that of normal group. However, although the microbial activity in the 480 mg/kg sodium propionate and 25% Sishen Pill + 240 mg/kg sodium propionate groups slightly increased relative to that in natural recovery group, these differences were not statistically significant (*p* > 0.05). These findings indicate that Sishen Pill have the potential to increase intestinal microbial activity in diarrhea mice with kidney-yang deficiency syndrome in a dose-dependent manner. On the other hand, sodium propionate did not significantly affect microbial activity in mice.

### 3.8 Effects of sodium propionate and Sishen Pill on the activity of intestinal enzymes in diarrhea mice with kidney-yang deficiency syndrome

**Figure 8:**
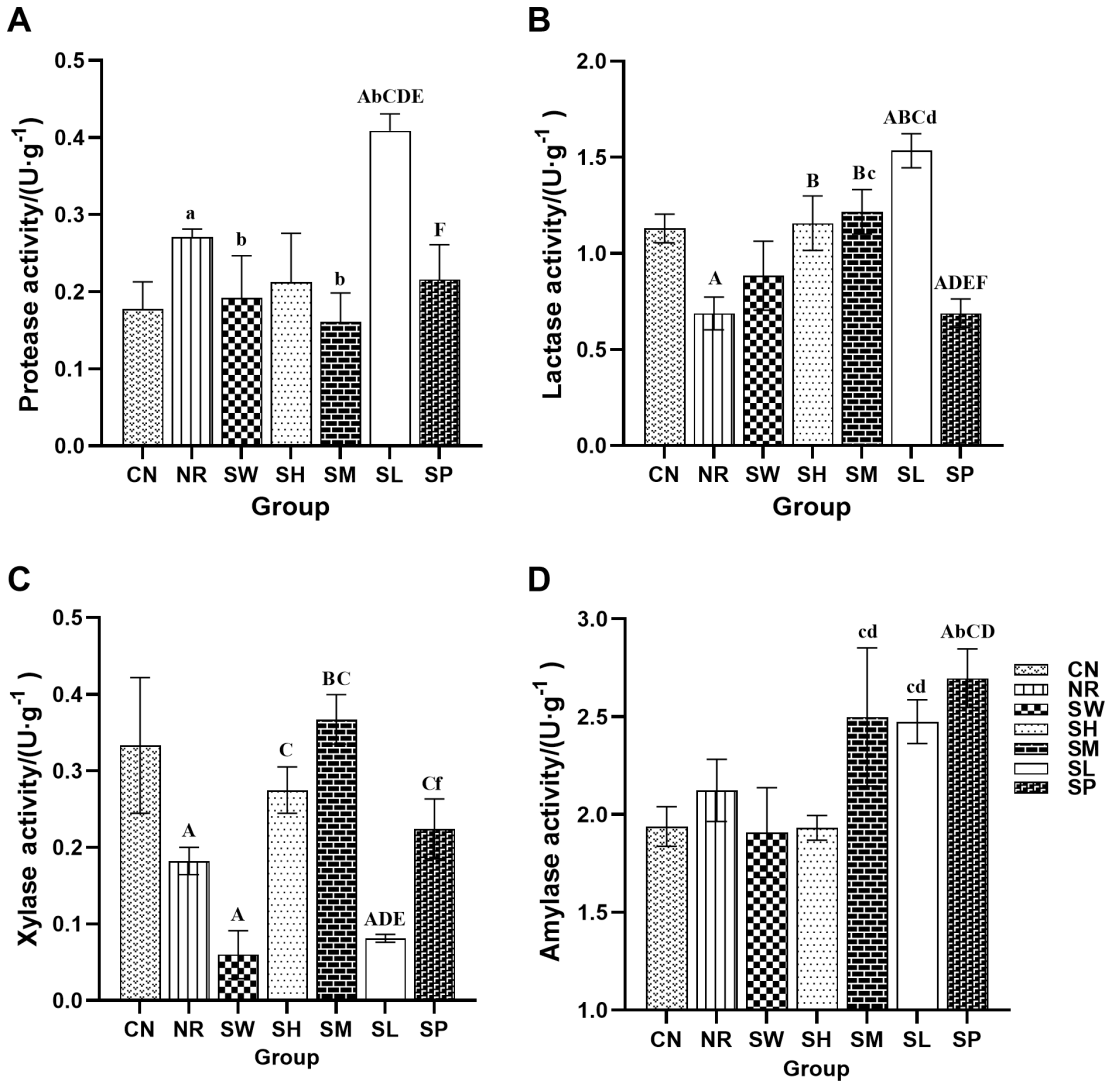
Effects of sodium propionate and Sishen Pill on the activity of intestinal enzymes in diarrhea mice with kidney-yang deficiency syndrome (x̄ ± s, N=6). Note: **(A)** Protease; **(B)** Lactase; **(C)** Xylanase; **(D)** Amylase.

As shown in **Figure 8**, the activity of proteases in the intestinal tract of the mice in natural recovery group was significantly greater than that in normal group (*p* < 0.05). In contrast, the activities of lactase and xylanase in natural recovery group were significantly lower than those in the normal recovery group (*p* < 0.01), while there was no significant difference in amylase activity between natural recovery group and normal recovery group (*p* > 0.05). These findings indicate that the modeling method can lead to an increase in intestinal protease activity while inhibiting the activity of lactase and xylanase and has no significant effect on amylase activity.

Compared with that in natural recovery group, the activity of intestinal protease in the 100% Sishen Pill and 50% Sishen Pill + 120 mg/kg sodium propionate groups was significantly lower (*p* < 0.05). Conversely, the protease activity in the 25% Sishen Pill + 240 mg/kg sodium propionate group was significantly greater than that in all the other groups (*p* < 0.01 or *p* < 0.05). These findings suggest that both the 100% Sishen Pill and 50% Sishen Pill + 120 mg/kg sodium propionate can reduce protease activity and restore it to normal levels, whereas 25% Sishen Pill + 240 mg/kg sodium propionate has the opposite effect on improving protease activity. Compared with natural recovery group, both the 75% Sishen Pill + 60 mg/kg sodium propionate group and the 50% Sishen Pill + 120 mg/kg sodium propionate group presented significantly increased intestinal lactase activity (*p* < 0.01). This finding indicates that the 75% Sishen Pill + 60 mg/kg sodium propionate and 50% Sishen Pill + 120 mg/kg sodium propionate effectively increase lactase activity, restoring it to normal levels, with the 25% Sishen Pill + 240 mg/kg sodium propionate demonstrating the most pronounced promoting effect. In terms of xylanase activity, the 50% Sishen Pill + 120 mg/kg sodium propionate group presented significantly greater levels than did natural recovery group (*p* < 0.01), whereas both the 100% Sishen Pill and 25% Sishen Pill + 240 mg/kg sodium propionate groups presented significantly lower levels than did normal group (*p* < 0.01). These results suggest that the 50% Sishen Pill + 120 mg/kg sodium propionate restored xylanase activity to normal levels, whereas both the 100% Sishen Pill and 25% Sishen Pill + 240 mg/kg sodium propionate had detrimental effects. The amylase activity of the 480 mg/kg sodium propionate group was significantly greater than that of the normal, natural recovery, 50% Sishen Pill + 120 mg/kg sodium propionate, and 25% Sishen Pill + 240 mg/kg sodium propionate groups (*p* < 0.01 or *p* < 0.05). These findings suggest that sodium propionate can increase amylase secretion within a specific dosage range.

## 4 Discussion

The literature indicates that adenine, as one of the commonly used substances for constructing the kidney-yang deficiency symptoms model, affects the energy metabolism of renal tissues, leading to pathological manifestations such as drop in body temperature, mental fatigue, a preference for quietness, and weight loss. *Folium sennae*, with its cold and bitter properties, can damage the spleen Qi when used excessively, impair the spleen Yang, and disrupt the spleen’s ability to transport and transform, ultimately resulting in a state of cold deficiency (Yanrong et al., 2021). The combination of adenine and *Folium sennae* is effective in establishing a model of diarrhea with kidney-yang deficiency syndrome, providing a more comprehensive and vivid representation of the clinical symptoms associated with this condition (Li et al., 2021b, 2015). Additionally, our study revealed that post-modeling, mice displayed behaviors such as arched backs, a propensity to huddle, cool bodies with cold tails, lethargy, soft stools that adhere readily to bedding, and the presence of fecal matter around the anal region. Moreover, the analysis of food intake, rectal temperature, body weight, and decreases in spleen and thymus indices further supported the successful establishment of the model (Guo et al., 2024).

Sodium propionate exerts a direct regulatory effect on the intestinal microbiota. Previous studies have demonstrated that the intake of sodium propionate significantly ameliorates vascular calcification in a rat model induced by vitamin D3 and nicotine (VDN) (Li et al., 2021a). Propionates are known to reduce intestinal pathogen penetration by enhancing the intestinal barrier, which helps preserve intestinal integrity against infections and inflammation (Fotschki et al., 2022). In the present study, The selected concentrations of sodium propionate (480 mg/(kg•d), 240 mg/(kg•d), 120 mg/(kg•d), and 60 mg/(kg•d)) are primarily based on pharmacokinetic considerations and previous published studies. The doses are chosen to reflect the physiological and therapeutic effects of sodium propionate while avoiding exceeding the toxicity threshold. By establishing a gradient from high to low doses, the dose-dependent effects can be effectively assessed. Moreover, the concentration range used in this study is consistent with those commonly used in previous research on sodium propionate and similar compounds. For example, published studies (e.g., Song et al., 2022, “Roseburia hominis increases gut melatonin levels and activates the p-CREB-AANAT pathway”) have shown that this dose range has been proven effective and safe in animal models and pharmacological studies, and is relevant to the diarrhea model used in this study (Song et al., 2022).

The administration of 480 mg/kg sodium propionate alone positively affected the condition of kidney yang deficiency, improved metabolic function, and increased blood circulation (Yan et al., 2022). Treatment with 100% Sishen Pill also facilitated the recovery of diarrhea mice with kidney-yang deficiency syndrome, as evidenced by the normalization of rectal temperature and body weight. These results are consistent with our previous findings, which showed that Sishen Pill had some effect on body weight and rectal temperature in mice with diarrhea with kidney-yang deficiency syndrome (Xie et al., 2024). However, the fecal water content in the 100% Sishen Pill group did not return to normal, possibly due to the relatively short duration of treatment (Chung et al., 2014). In recent years, the integration of TCA with microbiota-based interventions has become a prominent area of research. For example, a combination of 25% Qiwei Baizhu San ultramicro powder and 25% yeast has been shown to effectively treat dysbiosis-induced diarrhea in mice (Liang et al., 2012; Guo et al., 2015). Among the combinations tested, 75% Sishen Pill + 60 mg/kg sodium propionate group presented the most balanced therapeutic effect, effectively controlling diarrhea while promoting favorable body weight gain. These findings suggest a potential synergistic interaction between the two agents at this dosage, with sodium propionate enhancing the therapeutic efficacy of Sishen Pill in the treatment of diarrhea with kidney-yang deficiency syndrome. Although some improvements in the health status of the mice were detected in all the treatment groups, the therapeutic effects in the 25% Sishen Pill + 240 mg/kg sodium propionate and 50% Sishen Pill + 120 mg/kg sodium propionate groups were not significant. This lack of effect may be attributed to the insufficient dosage of Sishen Pill in these groups, which failed to achieve the desired therapeutic outcomes.

The organ index results further indicated that both Sishen Pill and 75% Sishen Pill + 60 mg/kg sodium propionate promoted the development of the spleen and thymus. Sishen Pill effectively alleviated the intestinal inflammatory response in diarrhea mice with kidney-yang deficiency syndrome, thereby supporting the health and function of immune organs (Li et al., 2023). Moreover, propionate has been shown to increase the number of regulatory T (Treg) cells, exerting balancing and regulatory effects on the immune system, as evidenced by the elevated thymus index (Schafer et al., 2021). Previous studies have demonstrated that the T-cell receptor (TCR) of Treg cells has a greater affinity for major histocompatibility complex (MHC) molecules in the thermic epithelial cells than conventional T cells do, suggesting stronger TCR specificity in Treg cells. This implies that Treg cells can bind with high affinity to T cells that have escaped negative selection, thereby exerting immunosuppressive effects (Miyara et al., 2014). However, the increase in the spleen index did not significantly correlate with this phenomenon, possibly due to the relatively limited responsiveness of the spleen to the adaptive effects of Treg cells. In 25% Sishen Pill + 240 mg/kg sodium propionate group and 50% Sishen Pill + 120 mg/kg sodium propionate group, no significant improvements in immune organ function were observed. This lack of effect may be attributed to an insufficient concentration of Sishen Pill, which may not have been adequate to effectively enhance immune organ function. Additionally, although the dosage of sodium propionate in these groups is relatively high, it may lead to immune system tolerance to sodium propionate, preventing it from achieving the expected immune-stimulating effect.

*Bifidobacteria* and *Lactobacillus* are widely recognized as beneficial for maintaining intestinal health and overall well-being, as they help preserve the balance of the intestinal microbiota (Azad et al., 2018). *Escherichia coli*, a facultative pathogen, is associated with diarrhea and inflammation (Yin and Wan, 2019). Small intestinal bacterial overgrowth (SIBO) is often considered a primary cause of chronic diarrhea and malabsorption (Bushyhead and Quigley, 2021). The genus *Bifidobacterium* is positively correlated with the levels of SCFAs, such as propionate, butyrate, and valerate (Zhou et al., 2023b). The 75% Sishen Pill + 60 mg/kg sodium propionate promoted the proliferation of *Bifidobacterium* in the intestines of diarrhea mice with kidney-yang deficiency syndrome while inhibiting the growth of harmful bacteria, including *E. coli*. This effect was greater than that of 100% Sishen Pill or 480 mg/kg sodium propionate treatments. This aligns with the effect of sodium propionate to promote the growth of beneficial bacteria and the role of Sishen Pill in modulating the intestinal microbiota (Abujamel et al., 2022). Together, these two treatments work synergistically to suppress the proliferation of pathogenic bacteria, and regulate the microbiota balance, laying the foundation for improving diarrhea associated with kidney-yang deficiency syndrome.

The activity of the intestinal microbiota can, to some extent, serve as an indicator of the overall metabolic capacity of the intestinal microbiome (Tang et al., 2020). This study revealed that 100% Sishen Pill, 75% Sishen Pill + 60 mg/kg sodium propionate, and 50% Sishen Pill + 120 mg/kg sodium propionate enhance the metabolic activity of the intestinal microbiota. In contrast, 480 mg/kg sodium propionate did not significantly affect microbiota activity, suggesting that the observed improvements in microbial activity are attributable primarily to Sishen Pill. These results align with the findings of Zhao et al., who reported that Sishen Pill exerts its therapeutic effects by reducing inflammation and modulating intestinal microbiota activity (Zhao et al., 2024).

Lactase plays a crucial role in the development of diarrhea. Research has demonstrated that congenital lactase deficiency can lead to severe osmotic diarrhea (Wanes et al., 2019). Amylase is predominantly secreted by the intestine itself, and any reduction in its activity can significantly impair the digestive and absorptive functions of the body (Wen et al., 2020). Xylanase, on the other hand, is primarily secreted by the intestinal microbiota (He et al., 2020). Michael et al. reported that proteases are involved in various biological inflammatory processes, particularly inflammation and tissue damage (Chen and Huang, 2024). Propionate, an active molecule, is capable of penetrating the cell wall of fungi, inhibiting intracellular enzyme activity, and consequently preventing the synthesis of β-alanine, thereby inhibiting fungal proliferation (Dijksterhuis et al., 2019). Sodium propionate, at specific dosages, has been found to inhibit lactase activity (Cherbut, 2003). Furthermore, a 25% sodium propionate solution has been shown to significantly increase the activities of trypsin and lipase (Wang et al., 2022). In the present study, a combination of 75% Sishen Pill and 60 mg/kg sodium propionate resulted in a reduction in protease activity, an increase in lactase activity, and alleviation of inflammatory responses, thereby improving diarrhea. Additionally, a combination of 50% Sishen Pill and 120 mg/kg sodium propionate significantly elevated both lactase and xylanase activities, thereby enhancing intestinal enzyme function. These findings suggest that sodium propionate, at doses of 60 mg/kg and 120 mg/kg, may facilitate the beneficial effects of Sishen Pill on intestinal enzyme activity. In contrast, the combination of 25% Sishen Pill with 240 mg/kg sodium propionate may not effectively suppress the release of proinflammatory factors. In some cases, it could even trigger a negative feedback mechanism, leading to a further increase in protease activity.

## 5 Conclusion

This study examined the therapeutic effects of various treatments on diarrheal mice with kidney-yang deficiency syndrome, including 100% Sishen Pill, 25% Sishen Pill + 240 mg/kg sodium propionate, 50% Sishen Pill + 120 mg/kg sodium propionate, 75% Sishen Pill + 60 mg/kg sodium propionate, and 480 mg/kg sodium propionate. The results revealed significant differences in therapeutic efficacy across the different treatment groups. Notably, the combination of 75% Sishen Pill with 60 mg/kg sodium propionate alleviated symptoms of diarrhea with kidney-yang deficiency syndrome, enhanced growth and development in the mice, suppressed excessive bacterial growth, promoted the proliferation of beneficial bacteria, and improved intestinal enzyme activity. This combination exhibited superior efficacy compared with either sodium propionate or Sishen Pill administered individually. These findings suggest that sodium propionate enhances the therapeutic action of Sishen Pill in the treatment of diarrhea with kidney-yang deficiency syndrome. However, further investigation is needed to determine whether this specific combination represents the most effective approach for treating diarrhea with kidney-yang deficiency syndrome.

## Data Availability

The raw data supporting the conclusions of this article will be made available by the authors, without undue reservation.
